# Zirconium preconcentration from zircon raffinate using gamma radiation–induced polymerization of reduced graphene oxide composite

**DOI:** 10.1007/s11356-023-26485-5

**Published:** 2023-03-29

**Authors:** Amr Hamdi Ali, Shaimaa Mohammed Abdo, Gehan Abdel Rahman Sadek Dakroury

**Affiliations:** 1 Nuclear Materials Authority, Maadi, P.O. 530, Cairo, Egypt; 2 Nuclear Chemistry Department, Hot Laboratories Centre, Egyptian Atomic Energy Authority, P.O. 13759, Cairo, Egypt

**Keywords:** Reduced graphene oxide, Polyacrylic acid composite, Zircon raffinate zirconium preconcentration, Sorption

## Abstract

Zirconium is commonly used as a cladding material for nuclear reactors. The purity of the zirconium material seeks to control reactor efficiency. A novel composite of reduced graphene oxide–grafted polyacrylic acid, malic acid, and trioctylamine (rGO-g-PAA-MA/TOA) was prepared using in situ radical polymerization with gamma radiation at a dose of 25 KGy from a ^60^Co cell to preconcentrate zirconium Zr(IV) from zircon raffinate. Five distinct rGO-g-PAA-MA/TOA composite compositions were created and evaluated. The best composite composition was 62.95% acrylic acid, 15.8% malic acid, and 15.8% trioctylamine. After 60 min, the sorption reaction reached equilibrium at pH 0.35 and 20 °C. The pseudo *n*^th^ order indicated that the order of the sorption reaction was 1.8476. The Elovich model and Dubinin-Radushkevich model controlled the kinetic mechanism and adsorption isotherm of the sorption reaction, respectively; based on estimated regression plots and quantitatively with three different error functions: coefficient of determination (*R*^2^), chi-square statistic (*χ*^2^), and corrected Akaike information (AIC_*c*_). The adsorption capacity of rGO-g-PAA-MA/TOA was 75.06 mg g^−1^. Exothermic reaction and spontaneous sorption took place. Using 2 M H_2_SO_4_, 98% of the zirconium was efficiently desorbed. The separation of contaminated Ti(IV) from desorbed Zr(IV) by raising pH to 2.5 through hydrolysis and ZrO_2_ formation.

**Supplementary Information:**

The online version contains supplementary material available at 10.1007/s11356-023-26485-5.

## Introduction

Zirconium is only found in its compounds in the IV oxidation state, and its properties are similar to those of titanium (IV). Zr(IV) and ZrO^2+^ ions exist in HNO_3_ and HC1O_4_ solutions and polymerize as zirconium can be formed with the fluoride ion, ethylene diamine tetraacetic acid, and hydroxy acids (Ali 2022). Zirconium has excellent thermal and mechanical properties, which make it suitable for many different applications like painting, glazes, dyes in the ceramic industry, refractory materials, smelting molds, and organic catalysts (Biswas et al. 2012; Rajmane et al. 2006). In addition to its application in the nuclear industry as a covering material for the fuel rods in nuclear reactors due to its low neutron capture cross-section, some zirconium compounds, such as zirconium phosphate and zirconium silico-tungstate, could be used to remove uranium from nuclear waste solutions (Ali 2018; Eliwa and Mubark 2021). Due to all of these applications in various fields, scientists are looking for various chemical techniques for extracting zirconium from its concentrates.

In nature, zirconium metal exists in two minerals: zircon (ZrSiO_4_) and baddeleyite (ZrO_2_) (Wang and Lee 2016; Wang et al. 2013). The zircon mineral is widely found in the ground along with igneous, metamorphic, and sedimentary rocks, as well as heavy residual rocks or beach sand. It should be separated from these admixtures using wet gravity concentration, followed by magnetic and electrostatic separation (Abdel-Rehim and Bakr 2016). For extracting ZrO_2_ from zircon minerals, the bonds between ZrO_2_ and SiO_2_ must be broken either chemically or thermally. Numerous methods were used to separate ZrO_2_ such as alkaline dissociation (Ma et al. 2020; Lötter et al. 2012), chlorination (Pechishcheva et al. 2018), reduction (Choi and Yoon 2013), hydrothermal treatments (Ianos and Barvinschi 2010; Taylor and Meyer 2005), spray pyrolysis (Rainer et al. 2022), zirconium oxychloride separation by crystallization (Poernomo et al. 2020), and precipitation (Ma et al. 2020; Chen et al. 2015; Ao et al. 2020; Gibot et al. 2020).

Numerous solvent extraction studies were carried out to investigate zirconium extraction from various solutions, such as organic phosphorus compounds such as TBP (Aliakbari et al. 2014), TOPO (Banda et al. 2013), TRPO (trialkylphosphine oxide) (Xu et al. 2012), CYANEX 301 and 302 (Saberyan et al. 2008, 2010), CYANEX 272 (Taghizadeh et al. 2009), CYANEX 923 (Taghizadeh et al. 2011), CYANEX 921 (El Shafie et al. 2014), amines (Lakshmanan et al. 2014), and D_2_EHPA (Taghizadeh et al. 2009; De Beer et al.^2016^). All these mentioned solvents showed good results for zirconium recovery. TOA (trioctylamine) especially has been used successfully to extract zirconium (Rajmane et al. 2006; Bhatta et al. 2013). However, the higher TOA concentration consumed, in addition to the contamination of Zr with Hf, is regarded as a weakness in this method. Organic acid media are known to provide better metal separation, possibly due to the high stability of metal–organic acid complexes (Rajmane et al. 2006; Van der Westhuizen et al. 2015). As a result, the authors are forced to look for a more cost-effective technique, such as adsorption to extract zirconium in pure form.

Until now, no papers have been published on the use of organic acids and TOA in a polymeric matrix to extract zirconium from its ore. Scientists became more interested in graphene, nanomaterials and carbon nanotubes as sorbent materials over the last decade (Valcárcel et al. 2008). Among them, graphene is preferred due to its low cost of preparation. Adding graphene to polymeric materials could improve their mechanical, thermal, and chemical properties, as well as their specific surface area (Trikkaliotis et al. 2021; Rostamnia and Pourhassan 2013; Alamgholiloo et al. 2021, 2020).

The current study focuses on the synthesis of a novel polymeric composite reduced graphene oxide–grafted poly acrylic acid, malic acid, and trioctylamine (rGO-g-PAA-MA/TOA) using gamma radiation for zirconium preconcentration and separation from zircon raffinate solution (the remained aqueous solution after solvent extraction). Following Egyptian zircon alkaline fusion and solvent extraction, the investigated composite was contacted with raffinate. The dimensions controlling the preconcentration and separation processes, like acidic media type, pH, adsorbent dose (V/m), zirconium initial concentration, shaking time, and temperature, were investigated. Elution studies were carried out to assess the feasibility of using this composite for zirconium preconcentration and separation. Other factors influencing zirconium elution from loaded adsorbents, such as eluant agent type and concentration, were investigated to choose the optimum parameters for zirconium elution, separation, and refining.

## Methodology

The Egyptian Nuclear Materials Authority provides zircon raffinate solution. HCl acid, NaOH, HNO_3_ acid, and H_2_SO_4_ acid were purchased from Merck, Germany. Acrylic acid (AA) with 99% purity was obtained from Elf Chem. Co, France. Loba Chemie India provided 99% pure malic acid (MA). Methylene bis-acrylamide (DAM) and extra-pure graphite powders were provided by Merck, Germany. BDH Chemicals, Ltd., England, produced potassium permanganate (KMnO_4_) and sodium nitrate (NaNO_3_). Alpha Co., India, also delivered hydrogen peroxide, while ascorbic acid was obtained from Bratachem Co., Egypt.

### Fusion of zircon mineral

S.1 represents the elementary analysis of the zircon ore (Ali 2018). About 250 g of zircon is heated to 650 °C in a stainless steel melting pot with 312 g of NaOH pellets; the resulting smelt is composed of water-soluble sodium silicate (Na_2_SiO_3_) and water-insoluble sodium zirconate (Na_2_ZrO_3_); this step is demonstrated by Eq. (1) (Ali 2022)

1 $${\mathrm{ZrSiO}}_{4}+4\mathrm{NaOH}\to {\mathrm{Na}}_{2}{\mathrm{ZrO}}_{3}+{\mathrm{Na}}_{2}{\mathrm{SiO}}_{3}+2{\mathrm{H}}_{2}\mathrm{O}$$

After cooling, sodium silicate was dissolved by mixing the smelt with 4 times its weight with double distilled water and stirred at 60 °C for 2 h. In this step, a large amount of Na_2_SiO_3_ was dissolved, and a small amount of Na_2_ZrO_3_ was hydrolyzed to hydrated zirconia species Eq. (2). (Ali 2022)

2 $${\mathrm{Na}}_2{\mathrm{ZrO}}_3+{\mathrm{nH}}_2\mathrm O\rightarrow{\mathrm{ZrO}}_{2.\;}(\mathrm n-1){\mathrm H}_2\mathrm O+2\mathrm{NaOH}$$

The mixture was filtered on hot, and about 20 g from the solid residual precipitate (Na_2_ZrO_3_) was then solubilized in excess conc. HCl to keep pH lower than 1 to avoid zirconium hydrolysis at 90 °C for 2 h as given in following Eq. (3) (Ali 2022) and then directly filtered, yielding zirconium chloride solution with the concentration (12.01 gL^−1^) and the solid residual, which mainly consisted of unreacted zircon, HfO_2_, TiO_2_, Al_2_O_3_, ThO_2_, and H_2_SiO_3_ (Mohamed and Daher 2002).

3 $${\mathrm{Na}}_{2}{\mathrm{ZrO}}_{3}+4\mathrm{HCl}\to {\mathrm{ZrOCl}}_{2}+2\mathrm{NaCl}+2{\mathrm{H}}_{2}\mathrm{O}$$

### Preparation of zirconium working solutions

Previously (Ali 2022; Bhatta et al. 2013), the prepared chloride solution was solvent extracted, the raffinate (aqueous remained solutions from a previous work) about 1 L was collected, and its zirconium content was determined and precipitated by ammonia solution. To prepare the required working concentrations, the precipitate was dried and divided before being dissolved in various acids such as conc. HCl and HNO_3_ to determine the best suitable working media.

### Preparation of (rGO-g-PAA-MA/TOA) sorbent

The prepared graphene oxide by the Hummers process (Zaaba 2017) was used as a precursor for the production of reduced graphene oxide (rGO). One gram of GO was reduced by 10 g of ascorbic acid to produce rGO (Andrijanto et al. 2016). Five different monomer compositions of rGO-g-PAA-MA/TOA composites are prepared (Table 1). A total of 0.1 g of rGO was dispersed in 20 mL distilled water and acrylic acid (AA) and magnetically stirred for 2 h at 20 °C, labeled mixture 1. Acrylic acid and 1 mL of trioctylamine were mixed in another 20 mL of distilled water and labeled mixture 2 after stirring for 2 h. Another 10 mL of distilled water was labeled solution 3; mixture of acrylic acid and L-malic acid was mixed and magnetically stirred for 2 h. Finally, with vigorous stirring, mixture 1 was added dropwisely to mixture 2 and then to mixture 3. MBA, a cross-linker was added, and the entire mixture was ultrasonically treated to form a homogeneous solution. S.2 illustrates a suggested mechanism of the polymerization process of rGO-g-PAA-MA/TOA.

**Table 1:** Composition of the monomers of (rGO-g-PAA-MA/TOA) composites

Sample	AA (Mol %)	MA (Mol %)	rGO (Mol %)	TOA (Mol %)	DAM (Mol %)
(rGO-g-PAA-MA/TOA)	62.95	15.80	1.50	15.80	3.95
(r_1_GO-g-PAA-MA/TOA)	47.25	31.50	1.50	15.80	3.95
(r_2_GO-g-PAA-MA/TOA)	31.50	47.25	1.50	15.80	3.95
(r_3_GO-g-PAA-MA/TOA)	39.37	39.37	1.50	15.80	3.95
(r_5_GO-g-PAA-MA/TOA)	15.80	62.95	1.50	15.80	3.95

### Instruments

A Bomen Miclson FT-IR spectrophotometer, model MB157 from Canada, was used to identify the active functional groups in rGO-g-PAA-MA/TOA composites. The crystalline phase structure was examined using Shimadzu X-ray diffraction (XRD) (model XD-Dl, Kyoto, Japan) with a diffraction angle (θ) range of 4–70°. For SEM and EDX mapping, a JEOL JSM-5400 (SEM, FEI Quanta FEG-250, and EDX) was used. Transmission electron micrograph image (TEM), type JEM2100, Jeol.s.b (Japan), is used to recognize the surface morphology and grain size of the prepared samples. A pore-size chromatech 9320 from the USA was used to calculate pore size distribution and porosity. The particle size is measured by Zetasizer Nano Zs, Malvern (UK), through the dynamic light scattering (DLS) method. BET surface area was measured according to the nitrogen BET (Brunauer–Emmett–Teller) adsorption technique in a surface area analyzer (Nova 3200 series, Micrometrics) (USA). Samples were degassed under a vacuum at 40 °C/4 h using the alizarin red S method; zirconium was analyzed in all aqueous phases (Pechishcheva et al. 2018; Elshehy 2011). The absorbance of the formed zirconium alizarin complex was measured at 520 nm against appropriate standard solutions using a Lambda 3 UV/VIS spectrophotometer (PerkinElmer, USA). The elementary concentration of leach liquor of zircon raffinate before, after sorption, and after desorption was measured by optical emission spectrometer with inductive coupled plasma (Prodigy Axial high dispersion ICP-OES model, USA).

### Batch experiments

Several influencing factors on the adsorption process were investigated. Media selection, solution pH, shaking time, initial zirconium concentration, adsorbent dose (V/m), and temperature were all considered. The adsorption experiments were repeated twice and sometimes three times by shaking 0.1 g sample portions of the prepared composite with 10 mL of zirconium concentrations ranging from 200–2000 mg L^−1^, pH ranging from 0.1 to 0.5, and at different temperatures [(20–60) ± 2] °C, and the samples were taken at predetermined intervals. Following treatment, the solid phases were separated using Whatmann filter paper (no. 40), and the amount of zirconium in the filtrate was determined using the alizarin red method. The adsorbed zirconium or uptake was calculated by dividing the difference between the initial and the residual concentrations in the solution by the mass of the adsorbent. The adsorption efficiency (% A) is determined as Zr(IV) adsorption percentage relative to its initial concentration of the system using Eq. (4) (Dakroury et al. 2021; Dolatyari et al. 2016).

4 $$\mathrm{Adsorption}\;\mathrm{efficiency}\;\mathrm{of}\;\mathrm{zirconium}(\mathrm A\%)=\left(\frac{{\mathrm C}_0-{\mathrm C}_{\mathrm e}}{{\mathrm C}_0}\right)\times100$$

Amount sorbed *q*_*e*_ (mg g^−1^) was calculated using Eq. (5)

5 $$q_e=\left({\mathrm C}_{\mathrm o}-{\mathrm C}_{\mathrm e}\right)\frac{\mathrm V}{\mathrm m}$$

where *q*_*e*_ (mg g^−1^) is the amount of the zirconium adsorbed per unit mass of adsorbent. *C*_*o*_ and *C*_*e*_ are the initial and equilibrium (or at any time) ion concentration (mg L^−1^), respectively, V is the volume of the solution per liter, and m is the mass (g) of the adsorbent.

### Zirconium desorption from loaded composite

Elution experiments were performed on loaded composite, in a 50-mL beaker, and 20 mL of eluant and 0.1 g of loaded composite were shaken vigorously using a magnetic stirrer for 24 h at 20 °C. A test portion of the aqueous samples was removed by pipette for analysis. The controlling factors of the elution process were investigated in detail, including eluant type and eluant concentration. The elution efficiency (%) was calculated using Eq. (6) (Ali et al.^2022^).

6 $$\mathrm{Desorption\%}=\frac{{C}_{\mathrm{aq}}}{{C}_{s}} \%$$

where *C*_aq_ is related to the concentration of zirconium within the aqueous phase and *C*_*s*_ is the concentration of zirconium within the composite.

### Kinetic and isothermal modeling

The interaction between rGO-g-PAA/MA/TOA and zirconium could be discussed through studying time transient models and equilibrium adsorption models. Pseudo-1st order, pseudo-2nd order, the Elovich, intraparticle diffusion models, and pseudo-*n*^th^ order were chosen for time transient models. Langmuir, Freundlich, and Dubinin-Radushkevich were selected for adsorption isotherm models. The non-linear regression was used to examine the selected applied models. S.3 includes non linear equations for the selected models. The best fit models were recognized qualitatively by residual error plots and quantitatively by three error functions: coefficient of determination (*R*^2^), chi-square statistic (*χ*^2^), and corrected akaike information (AIC_*c*_). S.4 includes the equation formula for the quantitative applied error functions. The best fitting model was accompanied by a maximum value of *R*^2^ or minimum values of *χ*^2^ and AIC_*c*_*.*

### Thermodynamic studies

Thermodynamic studies for zirconium sorption onto rGO-g-PAA-MA/TOA composite were conducted by considering four different temperatures (293, 303, 313, and 323 K), where all other key parameters (e.g., solution pH, sorbent concentration, and V/m ratio) were kept constant. Thermodynamic parameters, such as change in Gibbs energy (ΔG, kJ·mol^−1^), change in enthalpy (ΔH, kJ·mol^−1^), and change in entropy (ΔS, kJ mol^−1^·K^−1^), at the selected temperatures were calculated applying following Eqs. (7) and (8):

7 $$\Delta G^\circ=-RT\mathit\;ln\;K_c$$

8 $$ln\mathit\;K_c=\frac{\Delta S^\circ}R-\frac{\Delta H^\circ}{RT}$$

where *R* represents the general gas constant (8.314 JK^−1^ mol^−1^), *T* represents the absolute temperature (K), and *K*_*c*_ represents the sorption equilibrium constant.

## Results and discussion

### Characterization of rGO-g-PAA/MA/TOA Sorbent

#### FTIR analysis

**Fig.1:**
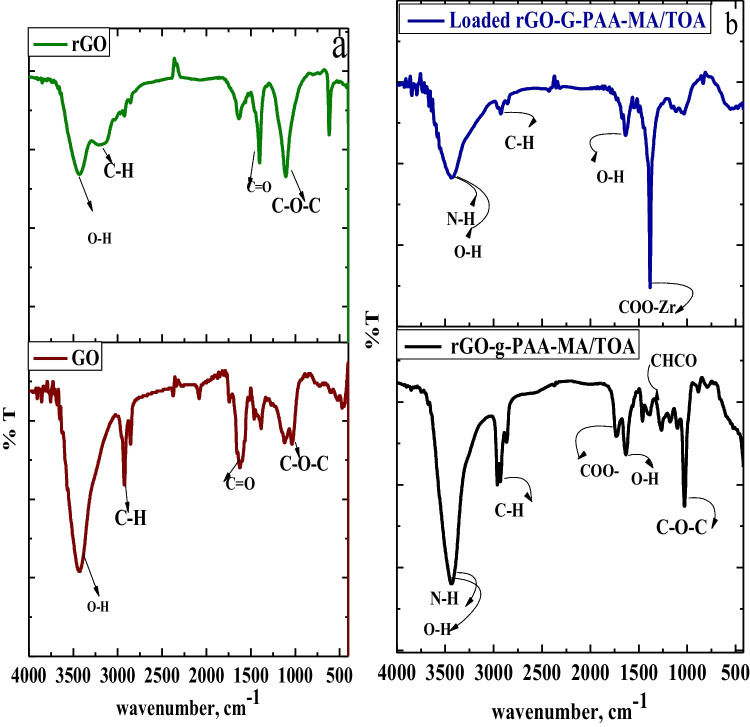
FT-IR of **a** GO (El-Shazly et al.^2022^) and rGO and **b** rGO-g-PAA-MA/TOA composite and Zr(IV) loaded onto rGO-g-PAA-MA/TOA composite

The graphene oxide (GO) FTIR spectrum in Fig. 1a investigates various oxygen derivative functional groups of GO such as O–H, C = O and C–O–C, as assigned by a strong wide peak at 3427 cm^−1^,1720 cm^−1^, and C–O–C, deformation by vibrations in epoxy groups (El-Shazly et al. 2022). After reduction of GO into rGO, the same functional groups were present, but the intensity of the oxygen derivatives functional groups decreased. Figure 1b depicts rGO-g-PAA/MA/TOA composite and loaded rGO-g-PAA/MA/TOA with Zr(IV), with the functional groups assigned. The integration of the O–H stretching vibrations of the grafted PAA with the N–H stretching vibrations of the TOA matrix results in a broadened absorption band at 3435 cm^−1^. The peak at 1733 cm^−1^ belongs to stretching, vibration of COO^−^. The two bands at 1461 cm^−1^ and 1386 cm^−1^ correspond to scissors and bending vibrations of CH_2_ and CHCO (Alvarez-Gayosso et al. 2015), respectively. The bands at 1633 cm^−1^ and 1230 cm^−1^ were related to the − OH bending of the neighboring carboxyl group. The peaks of C–H symmetrical and asymmetrical stretching of CH_2_ and CH_3_ composite are obtained at 2960 cm^−1^ and 2929 cm^−1^, where the vibration band at 884 cm^−1^ related to C–O–C deformation vibrations in the epoxy groups of rGO (Sieradzka et al. 2021). After Zr loading onto rGO-g-PAA/MA/TOA composite, some noticeable differences have been observed in the intensity of bands. Namely, the peak at 3437 cm^−1^, corresponding to the stretching vibrations of –OH and –NH_2_, became broader and of lower intensity, indicating these groups were involved in coordination of Zr(IV) ions. Disappearing of OH band at 1633 cm^−1^ and 1230 cm^−1^ and COO^−^ at 1730 cm^−1^ with a new high sharp intense band at 1403 cm^−1^ belongs to binding of Zr(IV) to OH^−^ and COO^−^ (Mizuguchi et al. 1997).

#### Morphology investigations

**Fig. 2:**
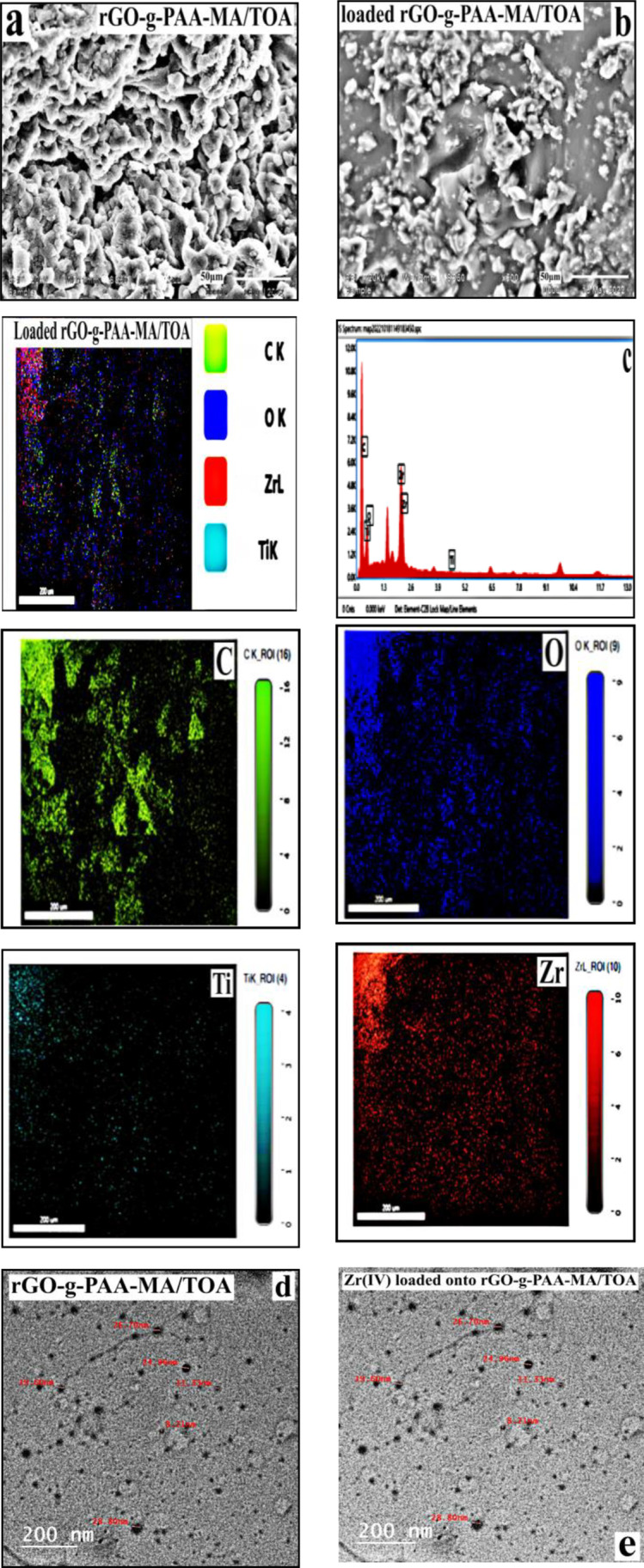
**a** SEM of rGO-g-PAA-MA/TOA composite and **b** SEM of loaded rGO-g-PAA-MA/TOA with Zr(IV) ions. **c** EDX mapping loaded rGO-g-PAA-MA/TOA with Zr(IV) ions. **d** TEM of rGO-g-PAA-MA/TOA composite. **e** TEM of loaded rGO-g-PAA-MA/TOA with Zr(IV) ions

Figure 2a reveals a rough, fractured, and randomly aggregated structure due to high cross-linking density of the rGO-g-PAA-MA/TOA composite (Czarnecka and Nowaczyk 2020). The surface particles are irregular in size as the hydrogen bonds between grafted AA and TOA lead to roughness in the surface (Li et al. 2021). The roughness surface guarantees reasonable sorption efficiency (Leiva et al. 2021). After Zr(IV) loading, Fig. 2b, the surface morphology became compactly packed and accumulated, with no discernible voids, indicating that Zr(IV) cations were entrapped via the active sites of rGO-g-PAA-MA/TOA. This was confirmed by the EDX mapping of loaded rGO-g-PAA-MA/TOA investigated in Fig. 2c where Zr, Ti was existed in addition to C, O the main elementary constituent of the composite. The presence of Ti is owed to the impurities in zircon raffinates (Ahmed et al. 2021). Figure 2d, e shows the TEM of rGO-g-PAA-MA/TOA and loaded rGO-g-PAA-MA/TOA where the nano-character was investigated before and after loading due to graphene content.

#### Porosity measurements and particle size distribution

**Fig. 3:**
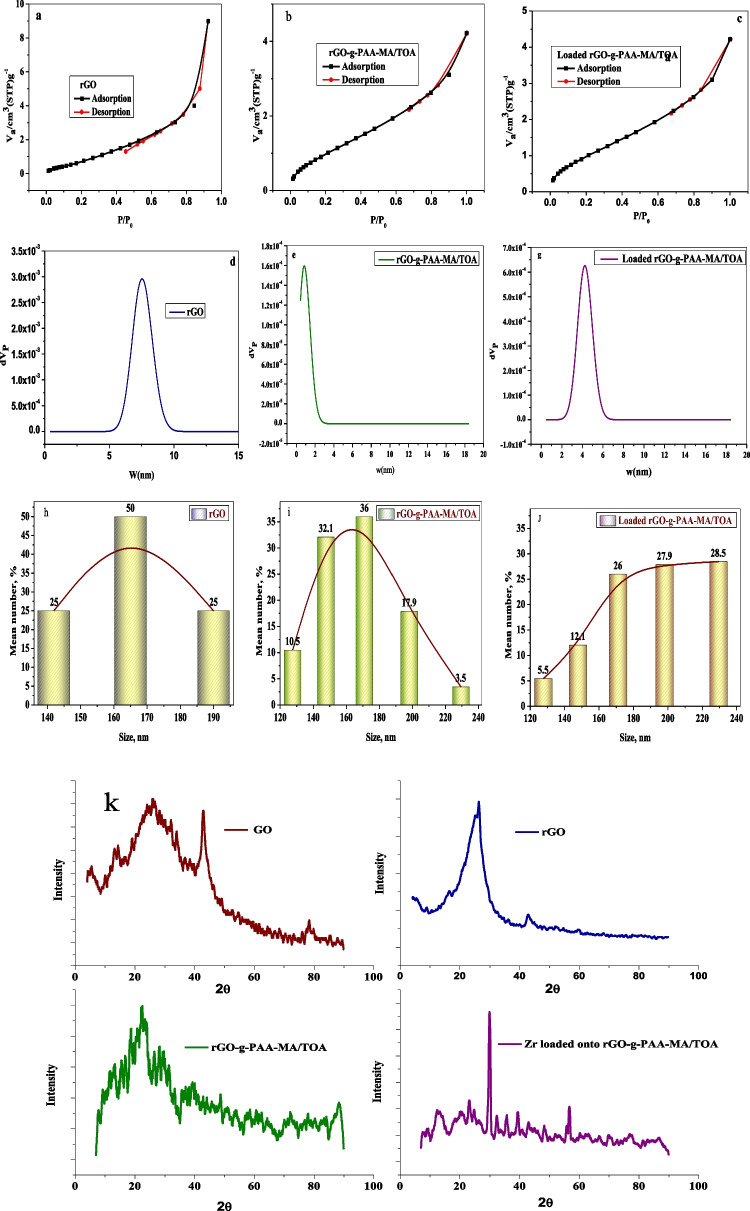
**a**–**c** BET isotherm of samples. **d**–**f** Pore size distribution. **h**–**j** Particle size distribution of rGO, rGO-g-PAA-MA/TOA composite, and loaded rGO-g-PAA-MA/TOA with Zr(IV) ions respectively. **k** XRD of GO, rGO, rGO-g-PAA-MA/TOA composite, and Zr(IV) loaded onto rGO-g-PAA-MA/TOA composite

Regarding to Fig. 3a–c, the BET adsorption isotherm of rGO, rGO-g-PAA-MA/TOA composite, and loaded rGO-g-PAA-MA/TOA composite suggesting type IV isotherm means mono-Zr(IV) molecule sorption. The hysteresis loops of type H_3_, characteristics of the mesoporous material with slit-shape pores or wedge-shaped pores according to IUPAC classification (Xu et al. 2020; Andrianainarivelo 2020) and monolayer formation, is beginning at low pressures, and multilayer formation occurs at medium pressure. The BET surface area was measured for rGO, rGO-g-PAA-MA/TOA composite, and loaded rGO-g-PAA-MA/TOA composite (0.2 g of the sample degassed at 40 °C). The BET surface area of rGO-g-PAA-MA/TOA composite was 4.56 m^2^ g^−1^ with meso-pore size. After loading, the specific surface area decreases to 3.76 m^2^ g^−1^ with meso-pore size due to the diffusion of Zr(IV) ions to pores. Pore size of rGO decrease after grafting to the polymeric matrix means that the composite material is much more selective (Andrianainarivelo 2020), while both the pore size and monolayer coverage (*V*_*m*_) of rGO-g-PAA-MA/TOA composite decrease after loading of Zr(IV) as shown in Table 2 and depicted in Fig. 3d, e, g confirming sorption process.

**Table 2:** Porosity measurements parameters of rGO-g-PAA-MA/TOA composite and loaded rGO-g-PAA-MA/TOA with Zr(IV) ions

	Parameter	Value
rGO	Total pore area (m^2^g^− 1^)	41.82
	Average pore diameter (nm)	50
	Bulk density (g mL^− 1^)	0.58
	Apparent density (g mL^− 1^)	0.83
	Porosity (%)	30.19
	BET—surface area (m^2^ g^− 1^)	5.37
	Mean pore diameter (BET nm)	10.352
	*V*_*m*_ (cm^3^(STP) g^− 1^)	1.23
rGO-g-PAA-MA/TOA	Total pore area (m^2 ^g^− 1^)	14.51
	Average pore diameter (nm)	139.60
	Bulk density (g mL^− 1^)	0.91
	Apparent density (g mL^− 1^)	1.67
	Porosity (%)	46.04
	BET—surface area(m^2^ g^− 1^)	4.56
	Mean pore diameter (BET nm)	5.56
	*V*_*m*_ (cm^3^(STP) g^− 1^	1.05
Loaded rGO-g-PAA-MA/TOA	Total pore area (m^2 ^g^− 1^)	7.122
	Average pore diameter (nm)	51.35
	Bulk density (g mL^− 1^)	0.25
	Apparent density (g mL^− 1^)	0.32
	Porosity (%)	22.46
	BET- surface area (m^2^ g^− 1^)	3.76
	Mean Pore diameter(BET nm)	4.57
	*V*_*m*_ (cm^3^(STP) g^− 1^)	0.86

Porosity measurements provide information about sorption efficiency. The average pore diameter of 139.5 nm represents macropore size (> 50 nm). These macropores enhance sorption and desorption efficiencies **(**Dakroury et al. 2022).

9 $$\mathrm{Bulk}\;\mathrm{density}=\frac{\mathrm{mass}}{\mathrm{sample}\;\mathrm{volume}+\mathrm{closed}\;\mathrm{pores}+\mathrm{open}\;\mathrm{pores}}$$

10 $$\mathrm{Apparent}\;\mathrm{density}=\frac{\mathrm{mass}}{\mathrm{sample}\;\mathrm{volume}+\mathrm{closed}\;\mathrm{pores}}$$

Only open pores play a role in the sorption process. Comparing apparent density and bulk density can provide insight into the extent of the open pores in the composite. All porosity measurements of the rGO and rGO-g-PAA-MA/TOA composite are shown in Table 2. A high percentage of porosity is compatible with roughness structure in the surface investigation of SEM results. After Zr(IV) loading onto rGO-g-PAA-MA/TOA composite, a decrease in total pore area, average pore diameter, and porosity was observed due to pore blockage with the larger Zr size species (Ali et al. 2011). The particle size of rGO, rGO-g-PAA-MA/TOA composite, and Zr loading onto rGO-g-PAA-MA/TOA composite is depicted in Fig. 3h–j where the average particle size increased after sorption of Zr onto rGO-g-PAA-MA/TOA.

#### X-ray diffraction

The structural phases of GO, rGO, rGO-g-PAA-MA/TOA composite, and Zr-loaded rGO-g-PAA-MA/TOA composite are depicted in Fig. 3k. A characteristic wide diffraction peak at 2 θ = 14.4° increasing the distance between fields in the graphene oxide (6.146 A°) is due to the presence of oxygen functional groups and water molecules into the carbon layer structure. After reduction of GO by ascorbic acid, the rGO structure became more regular. The characteristic diffraction peak of rGO at 2 θ = 24.3° with decreasing distance between layers (3.659 A°) referred to remaining oxygen functional groups (Johra et al. 2014). An amorphous structure assigned for rGO-g-PAA-MA/TOA composite is with a weak and broad diffraction characteristic peak of rGO at 2θ = 25–30° (Cao and Zhang 2015). Famous poor crystalline characteristic peaks for Zr(IV) appeared after Zr loading,at 2θ = 28.71, 31.76°, 35° (Devi and Jayashree 2013) confirming its sorption on the surface of rGO-g-PAA-MA/TOA composite.

### Sorption studies

Using the nitrate media and the chloride media, the sorption efficiency (%) and the sorbed amounts (mg g^−1^) for the five different compositions of the prepared composites were determined. The sorption efficiencies for all the prepared composites in the nitrate media were higher than those in the chloride media. The hard acid Zr(IV) (high charge density) binds to the hard base (Cl^−^) in the chloride media more than (NO_3_^−^) in the nitrate media, according to the (Pearson 1963) rule. So the sorption of Zr(IV) onto rGO-g-PAA-MA/TOA in the nitrate media was easier and higher than that in the chloride media. Table 3 demonstrates that rGO-g-PAA-MA-TOA with composition 62.95% AA had high sorption efficiency toward Zr(IV) when compared to the other prepared composite compositions. So the (rGO-g-PAA-MA/TOA) composite was selected for all sorption experimental studies during this work.

**Table 3:** The sorption efficiency (%) and sorbed amounts of P(rGO-g-PAA-MA/TOA) composite toward Zr(IV) from zircon raffinate

Sample	Nitrate media		Chloride media	
	Sorption efficiency (%)	Sorbed amount (mg g^−1^)	Sorption efficiency (%)	Sorbed amount (mg g^−1^)
(r_1_GO-g-PAA-MA/TOA)	39.96	56.9	15.45%	22
(r_2_GO-g-PAA-MA/TOA)	23.10	32.9	0.63%	9
(r_3_GO-g-PAA-MA/TOA)	37.71	53.7	0.77	11
(rGO-g-PAA-MA/TOA)	52.67	75	35.11%	50
(r_5_GO-g-PAA-MA/TOA)	17.91	25.5	0.28%	4

#### pH impact

**Fig. 4:**
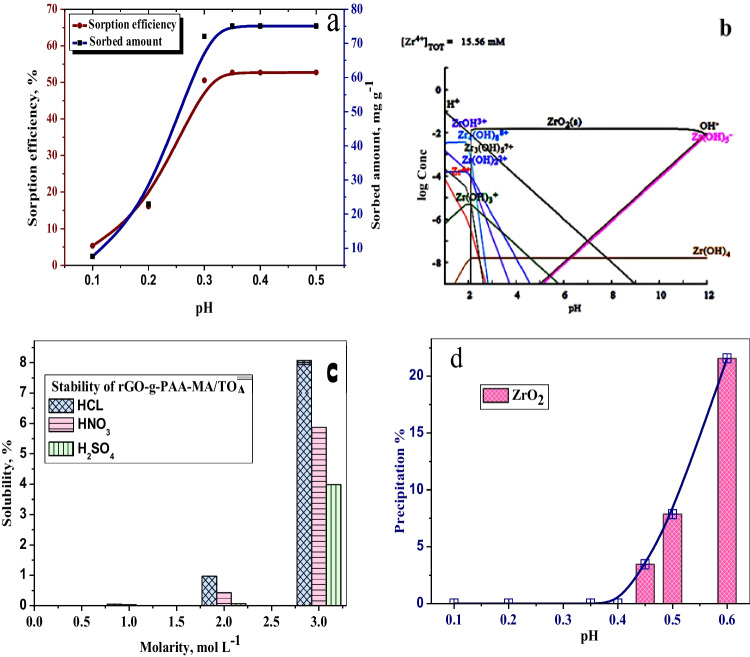
**a** Effect of pH on the sorption of Zr(IV) onto rGO-g-PAA-MA/TOA composite. **b** Speciation diagram. **c** Chemical stability of rGO-g-PAA-MA/TOA composite. **d** Precipitation curve of Zr(IV). (*C*_*i*_ = 1424.7 mg L^−1^, time = 24 h, temperature = 20 °C, V/m = 0.1L g^−1^)

In the nitrate media with pH ranging from 0.1 to 0.5, Zr(IV) sorption from zirconium raffinate is shown in Fig. 4a. The results revealed that increasing the pH value from 0.1 to 0.3 resulted in an increase in sorption efficiency, which was slightly affected by varying the pH within 0.3–0.5 range. Figure 4b depicts the speciation diagram for Zr at pH ranges 1–12 using Hydra/Medusa chemical equilibrium software (Puigdomenech 2013). At pH less than 2, complexion species such as [Zr(OH)_3_]^+^, [Zr(OH)_2_]^2+^, and [Zr(OH)]^3+^ and polymeric species [Zr_4_ (OH)_8_]^8+^ and [Zr_2_(OH)_6_]^2+^ predominate. An ionic species, Zr (OH)_5_, predominates at pH 5. Furthermore, Zr(IV) predominates at pH less than 1 (Christoph et al. 2017).

The stability of the rGO-g-PAA-MA/TOA composite was studied by measuring its solubility in HNO_3_, HCl, and H_2_SO_4_ (Fig. 4c). The results showed that the rGO-g-PAA-MA/TOA composite is chemically stable up to 2 M for all the examined mineral acids; the solubility of rGO-g-PAA-MA/TOA is less than 1%. The rGO-g-PAA-MA/TOA composite is soluble in HCl < HNO_3_ < H_2_SO_4_ at concentrations greater than 2 M. This is in agreement with the order of increasing the strength of acids, HCl < HNO_3_ < H_2_SO_4_. According to Fig. 4d, Zr(IV) began to precipitate at pH greater than 0.4. Thus, pH 0.35 is optimal for Zr(IV) sorption onto rGO-g-PAA-MA/TOA. Zr(IV) of high grade could be adsorbed onto rGO-g-PAA-MA/TOA at pH 0.35 and separated from Hf due to the formation of hafnium-polymeric species that inhibit its sorption onto the organic polymeric composite (Luo et al. 2016).

#### Time impact

**Fig. 5:**
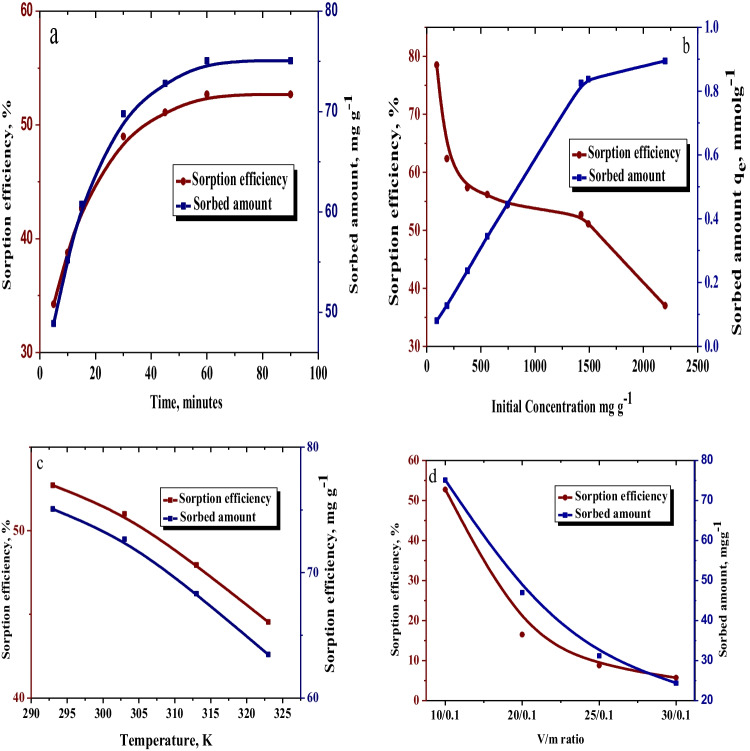
Effect of **a** time, **b** concentration, **c** temperature, and **d** V/m ratio on the sorption of Zr(IV) onto rGO-g-PAA-MA/TOA composite

The impact of time within the 5–90-min range and the equilibrium sorption time of zirconium onto rGO-g-PAA-MA/TOA composite at pH 0.35, with an initial zirconium concentration of 1424.7 mg L^−1^, are shown in Fig. 5a. Because of the presence of free active sites in the rGO-g-PAA-MA/TOA composite, the sorbed amount and sorption efficiency increase rapidly from 5 to 30 min. As these active sites occupied, the rate of sorption reaction slowed down at 30 min and reached equilibrium at 60 min. After the equilibrium time, there is almost no difference in sorption efficiency. A total of 75.06 mg g^−1^ of zirconium sorbed onto rGO-g-PAA-MA/TOA composite.

#### Concentration impact

The effect of concentration of zirconium on the extent of the sorption reaction is shown in Fig. 5b. The study of the effect of concentration performed within the range 93–2200 mg g^−1^ of zirconium. The sorption efficiency decreases with the increase in concentration due to the decrease in the available free active sites (Alghamdi et al. 2019) and the increase in the number of zirconium ions at composite surface due to driving force movement (Igberase et al. 2017). The sorbed efficiency of rGO-g-PAA-MA/TOA toward Zr(IV) decreased from 78.5% at initial concentration 93 mg L^−1^ to 37% at initial concentration 2200 mg L^−1^.

#### Temperature impact

Figure 5c implies a negative effect of the temperature on the sorption reaction at pH 0.35 for the initial concentration of 1424.7 mg L^−1^. As the temperature increased from 293 to 323 K, the sorption efficiency decreased from 52.7 to 44.5%. This behavior indicated that the Zr(IV) sorption reaction onto the rGO-g-PAA-MA/TOA composite was exothermic.

#### V/m ratio impact

A total of 0.1 g of rGO-g-PAA-MA/TOA composite is contacted with 10, 20, 25, and 30 mL of zirconium solutions to determine the optimum V/m ratio (Fig. 5d). The sorbed amount of zirconium and sorption efficiency decreased by increasing V/m ratio; this is because the number of free active sites on the rGO-g-PAA-MA/TOA composite is constant, while the number of zirconium ions increased with increasing the solution volume (Igberase and Osifo 2015).

### Time transient models

S.5a depicts the non-linear regression fitting plots of pseudo-1st order, pseudo-2nd order, the Elovich, and intraparticle diffusion models. The intraparticle diffusion clarified the external mass transfer of Zr(IV) to the external surface of rGO-g-PAA-MA/TOA composite as well as the internal diffusion of Zr(IV) to active sites. The calculated parameters and the applied error functions for the selected models are listed in Table 4. *R*^2^ for the Elovich was the highest one. As a result, the Elovich model provided the best fit model of Zr(IV) sorption onto the rGO-g-PAA-MA/TOA composite. The adsorption rate of the Elovich is higher than the desorption constant. A chemical adsorption or ion exchange mechanism of zirconium onto the rGO-g-PAA-MA/TOA composite was expected. Comparing pseudo-1st-order and pseudo-2nd-order parameters, the *q*_*t*_ calculated from pseudo-2nd order was 77.859 mg g^−1^ and closer to *q*_*t*_ (75.06 mg g^−1^) experiment, confirming that the sorption reaction is regulated by chemisorptions mechanism.

**Table 4:** Parameters and error functions data for time transient models studied for the sorption of Zr(IV) onto rGO-g-PAA-MA/TOA composite

Parameters	Time transient model
Pseudo-first order	
* q*_*t*_ (mg g^−1^) (calculated)	71.989
* K*_1_ (min^−1^)	0.175
* R*^2^	0.772
* χ*^2^	24.98
AIC_*c*_	34.171
Pseudo-second order	
* q*_*t*_ (mg g^−1^) (calculated)	77.859
* K*_2_ (g mg^−1^ min^−1^)	0.0037
* R*^2^	0.957
* χ*^2^	4.7345
AIC_*c*_	22.529
* q*_*t*_ (mg g^−1^) (experiment)	75.069
Elovich kinetic model	
* α* (mg g^− 1^ min^−1^)	289.678
* β* (g mg^−1^)	0.1006
* R*^2^	**0.962**
* χ*^2^	4.190
AIC_*c*_	21.674
Intraparticle diffusion model	
* k* (mgg^−1^ min^−1/2^)	3.749
* C* (mg g^−1^)	44.651
* R*^2^	0.8543
* χ*^2^	15.9915
AIC_*C*_	31.049
Pseudo *n*^th^ order	
* N*	1.8476
* k*_*n*_ (kg^n−1^ g^1 − n^ min^−1^)	7.6418 × 10 − 3
* R*^2^	0.928
* χ*^2^	**0.00139**
AIC_*C*_	** −36.042**

The highest *R*^2^ and the lowest *χ*2 and AIC_*c*_ are in bold

S.5b showed the plot of pseudo-*n*th order applying Eq. (11), which allows to describe the adsorption processes more accurately than using traditional pseudo-1st-order and pseudo-2nd-order equations. The calculated *n* order was 1.848; the order of the sorption of Zr(IV) onto rGO-g-PAA-MA/TOA composite was between 1 and 2; this pointed to multiple steps of the sorption reaction mechanism. The lowest values for *χ*^2^ and AIC_*C*_ were for pseudo-*n*th order.

Regarding the residual error in S.5c for the qualitative error function, positive deviations at the initial time are followed by negative ones at intermediate sorption time intervals and positive ones near equilibrium time for the Elovich model. Pseudo-*n*^th^-order model gives fairly lowest residuals.

### Isotherm modeling

Three famous isotherm models were examined: Langmuir, Freundlich, and Dubinin**-**Radushkevich. S.5d illustrated the predictive performance for non-linear regression of the examined isotherm models. The calculated parameters and quantitative error functions of the examined models are listed in Table 5. The D-R model has the highest *R*^2^ (0.894) and the lowest *χ*^2^ and AIC_*C*_ (0.0093 and − 30.696, respectively). Furthermore, the mean free energy *E* calculated from the parameter in Eq. 16 equals 8.168, confirming that the sorption process was chemisorption in accordance with the time transient results. For S.5e, the residual error of the fitting isotherm model showed that D-R has fairly low error residuals.

**Table 5:** Parameters and error functions data for adsorption isotherm models studied for the sorption of Zr(IV) onto rGO-g-PAA-MA/TOA composite

Parameters	Adsorption isotherm models
Langmuir model	
* q*_mL_	1.05159
* K*_L_	0.229
* R*_L_	0.806
* R*^2^	0.877
* χ*^2^	0.0109
AIC_*c*_	−29.449
Freundlich model	
* N*	2.234
* K*_*f*_ (mmol^n−1^ g^−1^ L -n)	0.2477
* R*^2^	0.771
* χ*^2^	0.0202
AIC_*c*_	−24.521
Dubinin-Radushkevich model	
* q*_mDR_ (mol g^−1^)	0.809
* β*_DR_ (mmol^2^ kJ^−^^2^)	7.493 × 10^−9^
* E* (kJ mol^−1^)	8.168
* R*^2^	**0.8945**
* χ*^2^	**0.0093**
AIC_*c*_	** −30.696**

The highest *R*^2^ and the lowest *χ*2 and AIC_*c*_ are in bold

### Thermodynamic studies

**Fig. 6:**
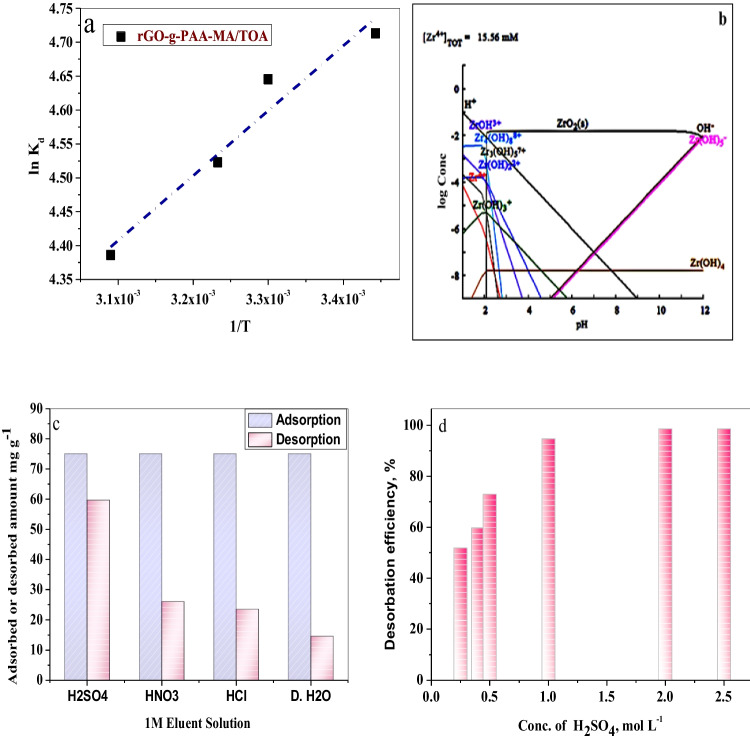
**a** Plot against ln *K*_*c*_ to 1/*T* for the sorption of Zr(IV) onto rGO-g-PAA-MA/TOA composite. **b** Speciation diagram of Zr(IV) contaminated with Ti(IV). **c** Desorption study of Zr(IV) sorbed onto rGO-g-PAA-MA/TOA composite using different eluents. **d** Desorption study of Zr(IV) sorbed onto rGO-g-PAA-MA/TOA using different concentrations of H_2_SO_4_

Plotting ln *K*_*c*_ against 1/*T* gave a straight line. The slope and intercept is used to calculate ∆H° and ∆S° at the selected temperatures. Figure 6a depicts the linear relation between ln *K*_*c*_ against 1/*T*. The calculated parameters are represented in Table 6. The negative value of the change in enthalpy indicates that the process is exothermic in nature, which can be substantiated by the decrease in sorption efficiency with an increase in temperature. On the other hand, the affinity and the increased randomness or disorders at the adsorbent-adsorbate interface during the period of sorption were corroborated by the positive value of ΔS (Sahmoune 2018). The ∆G° negativity increases with an increase in temperatures which indicates the spontaneity of the sorption reaction at higher temperatures.

**Table 6:** Thermodynamic parameters for Zr(IV) sorption onto rGO-g-PAA-MA/TOA composite

Composite	∆H (kJ mol^−1^)	∆*S* (J mol^−1^ K^−1^)	∆G (kJmol^−1^)			
			Temperature (K)			
			293	303	313	323
rGO-g-PAA-MA/TOA	−7.958	11.973	−11.47	−11.59	−11.71	−11.83

### Separation of zirconium from zircon raffinate

The prepared composite rGO-g-PAA/MA/TOA was used to separate Zr(IV) from zircon raffinate in the nitrate media at pH 0.35 by conducting 0.1 g of the composite with 10 mL of the raffinate liquor for an hour. The initial concentration of the elemental analysis of the zircon raffinate, after sorption onto rGO-g-PAA-MA/TOA composite and after desorption with 2 M H_2_SO_4_, was measured by ICP technique, and the results are listed in Table 7.

**Table 7:** Elemental analysis of initial, final Zircon Raffinate solution after sorption onto rGO-g-PAA-MA/TOA and desorbing solution of Zr-loaded rGO-g-PAA-MA/TOA

Element	Initial concentration (mg L^−1^)	Concentration after sorption (mg L^−1^)	Concentration after desorption (mg L^−1^)
ZrO_2_	932.75	680	660
HfO_2_	18.845	ND	ND
SiO_2_	464.167	0.03	< 0.01
Al_2_O_3_	0.85482	ND	ND
TiO_2_	0.71235	0.709	0.499
ThO_2_	0.285	ND	ND

*ND* not detected

The result showed that Ti(IV) was contaminated with desorbed Zr(IV), and to separate Ti(IV), the pH was raised to 2.5 where Zr(IV) and soluble cationic species of Zr hydrolyzed with the formation of ZrO_2_ (Fig. 6b) (Ahmed et al. 2021).

### Desorption studies

Desorption experiments were conducted to investigate the possibility of recovering adsorbed zirconium onto rGO-g-PAA-MA/TOA (Fig. 6c). To select the best desorbing agent, different eluents such as H_2_SO_4_, HNO_3_, HCl, and H_2_O were tested. The desorbing efficiency of zirconium using various eluents was in the order H_2_SO_4_ > HNO_3_ > HCl > H_2_O. So H_2_SO_4_ was selected as an efficient desorbing agent. Despite the fact that the order of *K*_*a*_ was in the order HCl > H_2_SO_4_ > HNO_3_ > H_2_O, the desorption efficiency order shows that H_2_SO_4_ is the best desorbing agent as discussed by Saberyan et al. (2010), while HCl and HNO_3_ were poor desorbing agents due to the common ion effect of Cl^−^ and NO_3_^−^, as HCl and HNO_3_ were used in fusion of Zircon minerals (Ali et al. 2022) and as pH media, respectively. Figure 6d shows the desorption efficiency of the different concentrations of H_2_SO_4_. As the concentration of H_2_SO_4_ increased from 0.25 to 2 M, the desorption efficiency increased from 51.81% H_2_SO_4_ to 98.6% at H_2_SO_4_.

### Reusability and mechanism of the sorption reaction

**Fig. 7:**
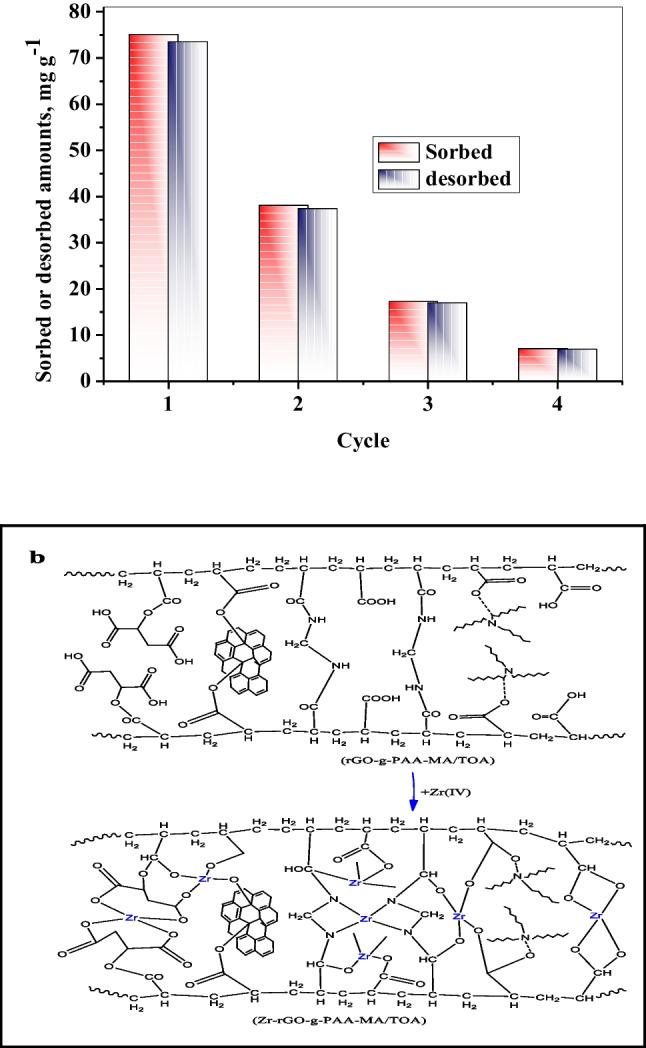
**a** Reusability of rGO-g-PAA-MA/TOA composite for adsorption of Zr(IV) using ([H_2_SO_4_] = 2 M). **b** Suggested mechanism of the Zr(IV) sorption reaction onto rGO-g-PAA-MA/TOA composite

To determine the ability of rGO-g-PAA-MA/TOA for recycling, four adsorption–desorption-adsorption cycles were carried out. The results of this study are investigated in Fig. 7a. The Zr(IV) sorption amount was 75 0.06 mg g^−1^ after the 1st cycle and decreased to 7.11 mg g^−1^ after the 4th cycle. It can be inferred that ion exchange or chemisorptions of Zr(IV) onto rGO-g-PAA-MA/TOA play a role in the sorption process (Ali et al. 2022).rGO-g-PAA-MA/TOA composite contains amine (− NH), hydroxyl (− OH), carbonyl (C = O), ether O–C–O, and ester (COO–) active groups, which can bind to Zr(IV) as shown in Fig. 7b for the predication of the sorption mechanism reaction where an electrostatic attraction between these groups and Zr(IV) has already been proposed. Furthermore, in addition to the electrostatic attraction force between Zr(IV) and active site functional groups, the intraparticle diffusion model plot of Zr(IV) into rGO-g-PAA-MA/TOA has a positive intercept of 44.651 mg g^−1^; thus, the diffusion process may be governed by both film diffusion and intraparticle diffusion together. Regarding to the above findings, the sorption mechanism of Zr(IV) onto rGO-g-PAA-MA/TOA, the preconcentration of Zr(IV) from zircon raffinate mechanism takes place through three routes: (i) movement of Zr(IV) from the solution bulk to rGO-g-PAA-MA/TOA surface, (ii) diffusion of Zr(IV) from boundary layer inside the pores of rGO-g-PAA-MA/TOA, and (iii) sorption of Zr(IV) onto active mentioned functional groups through electrostatic attraction force.

## Conclusion

Zirconium ions could be preconcentrated from zircon raffinate by sorption techmique, using a reduced graphene oxide–grafted polyacrylic-malic acid trioctylamine (rGO-g-PAA-MA/TOA) composite. Reduced graphene (rGO), acrylic acid (AA), malic acid (MA), and trioctylamine (TOA) monomers were co-polymerized by exposing the monomers to 25 KGy of cobalt 60 gamma-ray radiations. The sorption parameters were optimized to select pH 0.35, and sorption equilibrium was achieved after 1 h. The sorption efficiency decreased as the temperature increased, resulting in an exothermic sorption reaction. To achieve the sorption mechanism, kinetic modeling and isotherm modeling were used. Non-linear regression and three error functions were used to reduce fitting errors and select the best fitting mechanism for the sorption reaction. Coefficient of determination (*R*^2^), chi-square statistic (*χ*^2^), and corrected Akaike information (AIC_*c*_) were used as quantitative error functions, and residual plots were used as qualitative error functions. Thus, the Elovich regulated the reaction kinetic mechanism for kinetic models and the Dubinin-Radushkevich controlled the adsorption isotherms. The capacity for sorption was 75.06 mg g^−1^. Thermodynamic calculations clarified that the spontaneity of the sorption reaction produced positive entropy. Ninety-eight percent of the sorbed zirconium recovered using 2 M H_2_SO_4_ as an efficient desorbing eluent. The research demonstrated the potential of rGO-g-PAA-MA/TOA as a promising composite for the separation of Zr(IV) from Hf(IV) from zircon raffinate.

## Supplementary Information

Below is the link to the electronic supplementary material.

Supplementary file1 (DOCX 397 KB)

## Data Availability

All the data used for this work are publicly available.
