# Supplementary material for: Zirconium preconcentration from zircon raffinate using gamma radiation–induced polymerization of reduced graphene oxide composite
Source: Environ Sci Pollut Res Int. 2023 Mar 29;30(20):58330–45. doi: 10.1007/s11356-023-26485-5 (PMC10163083; doi:10.1007/s11356-023-26485-5)
Supplement: Supplementary file 1 — Supplementary file1 (DOCX 397 KB) [file 11356_2023_26485_MOESM1_ESM.docx]

**List of Supplementary 2**

**S.1.** Elementary analysis of zircon mineral (Ali 2018)

| **Compound Weight %** | **ZrO_2_ + HfO_2_** | **SiO_2_** | **Fe_2_O_3_** | **TiO_2_** | **MgO** | **CaO** | **Al_2_O_3_** | **K_2_O** | **Na_2_O** | **P_2_O_5_** |
| --- | --- | --- | --- | --- | --- | --- | --- | --- | --- | --- |
| **Zircon mineral** | 66.62 | 32.53 | 0.14 | 0.22 | 0.02 | 0.01 | 0.06 | < 0.01 | < 0.01 | 0.13 |

**S.2.** Suggested mechanism of the polymerization of rGO-g-PAA/MA/TOA composite

**S.3.** Non-linear form equations of Time transient models and equilibrium adsorption models

| **Model** | | **Non-linear form** | |
| --- | --- | --- | --- |
| **Time Transient models** | **pseudo 1st order**  (Dakroury and Abo-Zahra 2020) | $q_{t}=q_{e}\left( 1-e^{-K_{1}t} \right)$ (7) | |
|  | **pseudo 2nd order (**Dakroury et al. 2022a) | $q_{t}=\left( {(K}_{2}q_{e)}^{2}t) /(1+ K_{2}q_{e}t) \right)$(8) | |
|  | **Elovich model** (Kisiela-Czajka, and Dziejarski 2022)  **Intraparticle diffusion** (Eliwa et al. 2022) | $q_{t}= 1/\beta\ln(1+\alpha\beta t)$ (9)  $q_{t}=k_{i}t^{0.5}+ C$ (10) | |
|  | **Pseudo nth order**  (Tseng et al. 2014) | $\frac{\boldsymbol{q}_{\boldsymbol{t}}}{\boldsymbol{q}_{\boldsymbol{e}}}\boldsymbol{=1-}\frac{\boldsymbol{1}}{{\boldsymbol{[1+}\left( \boldsymbol{n-1} \right)\boldsymbol{q}_{\boldsymbol{e}}^{\boldsymbol{n-1}}\boldsymbol{k}_{\boldsymbol{n}}\boldsymbol{t]}}^{\boldsymbol{(}\frac{\boldsymbol{1}}{\boldsymbol{n-1}}\boldsymbol{)}}}$ (11) | |
| **Equilibrium adsorption models** | **Langmuir(**Dakroury et al 2022b) | $q_{e}= \left( q_{m}b C_{e} /1+bC_{e} \right)$(12) |  |
|  | **Freundlich** (Dakroury et al 2022b) | $q_{e}= K_{f} C_{e}^{1/n}$ (13) |  |
|  | **Dubinin-Radushkevich model** (Kisiela-Czajka, and Dziejarski 2022) | $q_{\mathrm{mDR}}=e^{-\beta_{\mathrm{DR}}\varepsilon^{2}}$ (14)  $\varepsilon=RT\ln( 1+ 1/C_{e} )$ (15)  E=$1/\sqrt{2\beta}$ (16) |  |

q_e_ and q_t_ are the sorbed amounts of zirconium (mg g^-1^) at equilibrium time and at any time t, respectively; k_1_ (min^-1^) is the pesudo 1^st^ rate constant, k_2_ (g mg^-1^ min^-1^) is the pseudo 2^nd^ order rate constant, α, β are the Elovich constants. is the sorption free energy constant C_e_ is the zirconium α is the Elovich initial adsorption rate (mg/g min), β (g/mg) desorption constant. K_i_ interparticle diffusion constant. n is the order of reaction, k_n_ rate constant of pseudo n^th^ order reaction. q_m_ is the monolayer sorption capacity (mg g^-1^), b concentration at equilibrium. K_f_ denotes for Freundlich constants, and n denotes for sorption capacity and intensity. q_mDR_ is monolayer capacity For D-R model, β_DR_ is a constant related to apparent adsorption energy, ε is polanyi potential, , R is the universal gas constant (8.314 J K^-1^ mol^-1^), and T is the absolute temperature (K).

**S.4.** Equations of applied quantitative error function (Kisiela-Czajka, and Dziejarski 2022)

| **Error Function** | **Equation** |
| --- | --- |
| **Coefficient of determination (R2)** | $R^{2}=1-\sum_{i=1}^{n} {(q_{calc.}-q_{exp.})}^{2}/\sum_{i=1}^{n} {(q_{calc.}-q_{mean})}^{2}$ (17) |
| **Chi-square statistic (𝛘2)** | $\sum\frac{{(q_{calc.}-q_{exp..})}^{2}}{q_{calc.}}$ (18) |
| **corrected Akaike information (AIC_c_)** | AIC_c_ =$\left( n\ln\left( SER/n \right) \right)+ \left( 2\left( p+1 \right) \right)+\left( \frac{2\left( p+1 \right)\left( p+2 \right)}{n}-p-2 \right) (19)$ |

q_exp_ (mg g^-1^) is the sorbed amount of zirconium, q_calc_. (mg g^-1^) is the predicted sorbed amount, *q*_mean_ (mg g^-1^) is the mean of *q*_exp_ values of zirconium, n is the experimental data points number, p modelling parameter number.

**S.5. a-** non linear fitting of kinetic modelling b- non linear fitting of pseudo n^th^ order

c - Residual plots of kinetic modelling. d- non linear fitting of Isotherm modelling

e - Residual plots of Isotherm modelling for the sorption of Zr(IV) onto rGO-g-PAA-MA/TOA composite.

References

Dakroury GA, Abo-Zahra SF (2020) The use of titanium oxide/polyethylene glycol nanocomposite in sorption of Cs and Co radionuclides from aqueous solutions. J Radioanal Nucl Chem 324:1351–1364. https://doi.org/10.1007/s10967-020-07167-9

Dakroury GA, El-Shazly EAA, Hassan HS (2022) Sorption of lead (II) and strontium (II) ions from aqueous solutions onto nonliving Chlorella vulgaris alga/ date pit activated carbon composite. Carbon Lett 32:495–512. https://doi.org/10.1007/s42823-021-00280-z

Eliwa AA, Mubark AE, Dakroury GA, Ehab AA, El-Azony KM (2022) Polyacryl-dimethyl-heptadecanamine-mullite as a

promising sorbent for chromium and vanadium sorption from ilmenite. J Environ Chem Eng 10(6):2213–3437.

https://doi.org/10.1016/j.jece.2022.108886

Ibrahim HA, Abdel Moamen OA, Monem NA, Ismail IM (2018) Assessment of kinetic and isotherm models for competitive

sorption of Cs and Sr from binary metal solution onto nanosized zeolite. Chem Eng Commun 205:1274–1287

Jang Y-J, Liu S, Yue H et al (2020) Hydrophilic biocompatible poly (acrylic acid-co-maleic acid) polymer as a surface-coating ligand of ultrasmall Gd O nanoparticles to obtain a high r1 value and T1 MR images. Diagnostics 11:2

Kisiela-Czajka AM, Dziejarski B (2022) Linear and non-linear regression analysis for the adsorption kinetics of SO in a fixed carbon bed reactor—a case study. Energies 15:633. https://doi.org/10.3390/en15020633

Kumar KV, Porkodi K (2007) Mass transfer, kinetics and equilibrium studies for the biosorption of methylene blue using Paspalum notatum. J Hazard Mater 146:214–226. <https://doi.org/10.1016/j.jhazmat.2006.12.010>

Tseng R-L, Wu P-H, Wu F-C, Juang R-S (2014) A convenient method to determine kinetic parameters of adsorption processes by nonlinear regression of pseudo-nth-order equation. Chem Eng J 237:153–161. https://doi.org/10.1016/j.cej.2013.10.013
